# Simultaneous Source Localization and Polarization Estimation via Non-Orthogonal Joint Diagonalization with Vector-Sensors

**DOI:** 10.3390/s120303394

**Published:** 2012-03-08

**Authors:** Xiao-Feng Gong, Ke Wang, Qiu-Hua Lin, Zhi-Wen Liu, You-Gen Xu

**Affiliations:** 1 School of Information and Communication Engineering, Dalian University of Technology, No. 2 Linggong Road, Dalian 116024, China; E-Mails: ke14011722@qq.com (K.W.); qhlin@dlut.edu.cn (Q.-H.L.); 2 School of Information and Electronics, Beijing Institute of Technology, 5 South Zhongguancun Street, Beijing 100081, China; E-Mails: zwliu@bit.edu.cn (Z.-W.L.); yougenxu@bit.edu.cn (Y.-G.X.)

**Keywords:** complex non-orthogonal joint diagonalization, direction-of-arrival, polarization, electromagnetic vector-sensor, LU, LQ

## Abstract

Joint estimation of direction-of-arrival (DOA) and polarization with electromagnetic vector-sensors (EMVS) is considered in the framework of complex-valued non-orthogonal joint diagonalization (CNJD). Two new CNJD algorithms are presented, which propose to tackle the high dimensional optimization problem in CNJD via a sequence of simple sub-optimization problems, by using LU or LQ decompositions of the target matrices as well as the Jacobi-type scheme. Furthermore, based on the above CNJD algorithms we present a novel strategy to exploit the multi-dimensional structure present in the second-order statistics of EMVS outputs for simultaneous DOA and polarization estimation. Simulations are provided to compare the proposed strategy with existing tensorial or joint diagonalization based methods.

## 1. Introduction

**Figure 1.**
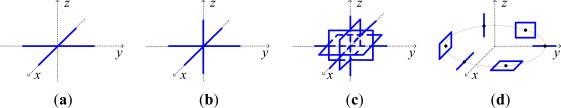
An illustration of four typical EMVS’s. (**a**) Cross-dipole. (**b**) Tripole. (**c**) The cocentered complex EMVS. (**d**) The distributed complete EMVS.

An electromagnetic vector-sensor (EMVS) comprises 2∼6 electromagnetic (EM) sensors (e.g., orthogonally oriented short dipoles and small loops arranged in a co-located or distributed manner, see Figure 1) that provide complete or partial measurements of the EM fields induced by the incident sources [1–5]. As a result, using EMVS in direction finding and polarization estimation could yield additional advantages over the conventional scalar sensor array processing techniques, mainly due to the fact that the distinction of impinging sources could be well exploited in both the spatial and polarization domains by using EMVS arrays. Particularly, in the early works on direction finding and polarization estimation with EMVS’s, maximum likelihood (ML) [3,6,7], multiple signal classification (MUSIC) [8–10], subspace fitting [11,12], and estimation of signal parameters via rotational invariance techniques (ESPRIT) [13–18] have been derived from their scalar array based counterparts, and some other issues such as vector cross-product based source tracking [4,19,20], polarimetric smoothing for coherent source localization [21–24], the analysis of performance [25,26] and identifiability [27–31] were also addressed.

The early works unfolded the potential of using EMVS’s in array processing through both analysis and experiments. However, there was still a problem as these works rarely considered the multi-dimensional (MD) data structure of the EMVS array outputs in the time-space-polarization domains. More exactly, the ML and MUSIC variants usually concatenate the vectorial output from each EMVS into a long-vector, which somehow undermines the MD structure of the array outputs. The ESPRIT variants, on the other hand, did consider this MD structure of array outputs in the form of multiple rotational invariant matrix slices, yet only used these matrix slices in a pairwise manner such that only a fraction of the MD structure is exploited each time. As such, the use of higher-dimensional algebras such as tensors and hypercomplex has attracted increasing attention in the recent years [32–44], for a better exploitation of the afore-mentioned MD structure present in the EMVS signals. More exactly, tensors are employed to model the MD structure of EMVS array signals as a multilinear algebraic quantity, upon which parallel factor analysis (PARAFAC) and higher-order singular value decomposition (HOSVD) are used to exploit this presented multilinearity [32–37]. Hypercomplexes, on the other hand, handled the above-mentioned MD structure by encapsulating the local vectorial output of each EMVS into a multinion (e.g., quaternion, biquaternion, quad-quaternion, bicomplex, or a more generalized geometric algebra model) [38–43], of which multiple imaginary parts are used and defined under certain hypercomplex algebraic rules. Recently, there have also been works on combining tensor decompositions and hypercomplex operations [44]. It is demonstrated in these works that an efficient exploitation of the MD structure for EMVS array signals could bring out improved performance over the conventional methods, with regards to the robustness to the errors introduced by noise, finite data length, and model errors.

In this paper, we will introduce an alternative strategy to tensor and hypercomplex based methods, for exploiting the MD structure of the outputs for an array of six-component cocentered complete EMVS’s (for clarity hereafter we name it EMVS without causing any misunderstanding), in the context of joint DOA and polarization estimation. More specifically, we formulize the MD structure of an EMVS array outputs as a set of complex square matrices that share a jointly diagonalizable structure, and propose to fit this structure by complex-valued non-orthogonal joint diagonalization (CNJD) for simultaneous DOA and polarization estimation. Moreover, we consider the LU or LQ decompositions for the target matrices and formulize the optimization problem in CNJD as two alternating stages: the L-stage and U(Q)-stage. In addition, inspired by the Jacobi-type schemes for joint diagonalization, we further replace the sub-optimization problem in each of the above two stages by a sequence of elementary rotation matrices which rely on only one or two parameters, and propose two closed-form CNJD algorithms.

The rest of the paper is organized as follows: Section 2 presents the data model of an EMVS array, and its formulation into CNJD problems. Section 3 introduces three proposed CNJD algorithms and the corresponding joint DOA and polarization estimation strategy, as well as some implementation remarks. Section 4 provides simulations to compare the proposed strategy with some existing tensorial methods to illustrate the potential of the proposal. Section 5 concludes the manuscript.

## 2. Measurement Model and Problem Formulation

### 2.1. Measurement Model of an EMVS Array Output

**Figure 2.**
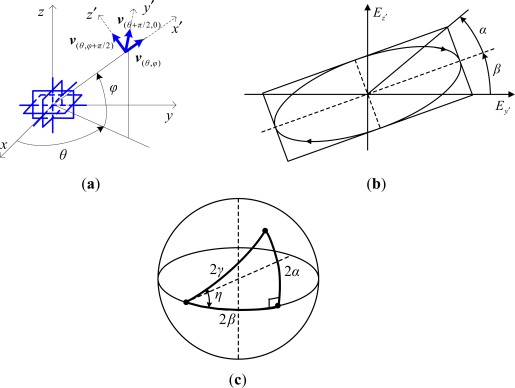
The angle and polarization definitions. (**a**) The angle definition. (**b**) Polarization ellipse. (**c**) Poincare sphere.

Let (*θ*,*φ*) be the azimuth-elevation 2D DOA of a narrow-band planar EM signal (see Figure 2(a) for the angle definition). The induced electric field vector of this signal moves along the polarization ellipse which could be characterized by the ellipse angle *α* and orientation angle β, and the polarization state could further be represented by (*γ*,*η*) that are linked to (*α*,*β*) in the Poincare sphere [1] (see Figure 2(b,c) to visualize the polarization ellipse and the relationship between(*γ*,*η*) and (*α*,*β*)), 0 < *θ* < 2π, |*φ*| ≤ π/2, 0 < *γ* < π/2, |*η*| ≤ π. The output of an EMVS is written as [2]:

(1) $$p_{\theta ,\varphi ,\gamma ,\eta }≜\left[\begin{array}{c} e_{\theta ,\varphi ,\gamma ,\eta }\\ h_{\theta ,\varphi ,\gamma ,\eta } \end{array}\right]=\left[\begin{array}{cc} v_{\left(\theta +\pi /2,0\right)} & -v_{\left(\theta ,\varphi +\pi /2\right)}\\ v_{\left(\theta ,\varphi +\pi /2\right)} & v_{\left(\theta +\pi /2,0\right)} \end{array}\right]\;\left[\begin{array}{c} \text{cos}\;\gamma \\ \text{sin}\;\gamma e^{i\eta } \end{array}\right]$$

where ***e***_*θ*,*φ*,*γ*,*η*_ ∈ C^3^ and ***h***_*θ*,*φ*,*γ*,*η*_ ∈ C^3^ are the collected electric and magnetic field vectors, respectively. ***v***_(*θ*, *φ*)_ ≜ [cos *θ* cos *φ*, sin *θ* cos *φ*, sin *φ*]*^T^* denotes the Poynting vector containing the three coordinates associated with orientation (*θ*,*φ*). Therefore, ***v***_(*θ*,*φ*)_, ***v***_(*θ+π/2*,0)_ and ***v***_(*θ*,*φ+π/2*,0)_ constitute a mutually orthogonal triad as shown in Figure 2(a). We herein label ***p***_*θ*,*φ*,*γ*,*η*_ as the angular-polarization steering vector as it reflects the amplitude-phase relationship among the received EM fields, which depends on both DOA and polarization.

In addition to the unit-EMVS formulization in Equation (1), we further assume the receiving antenna array comprises *N* EMVS’s. The position coordinates of the *nth* EMVS are encapsulated in a vector *k_n_* ∈ R^3^, *n* =1, 2, …, *N*. As a result, the phase delays of all the EMVS’s with regards to the one at the origin could be characterized by the so-called spatial steering vector defined as follows:

(2) $$d_{\theta ,\varphi }≜\text{exp}(\vec{i}2\pi \lambda^{-1}{\left[k_{1}^{T}v_{\left(\theta ,\varphi \right)},k_{2}^{T}v_{\left(\theta ,\varphi \right)},...,k_{N}^{T}v_{\left(\theta ,\varphi \right)}\right]}^{T})$$

where λ is the wavelength of the impinging signal, and i⃗ denotes the imaginary unit. As a result, the EMVS array output for a signal with DOA (*θ*,*φ*) and polarization (*γ*,*η*) is given by:

(3) $$X_{\theta ,\varphi ,\gamma ,\eta }(t)≜p_{\theta ,\varphi }\;d_{\theta ,\varphi ,\gamma ,\eta }^{T}\;s(t)$$

where *s*(*t*) is the complex envelop of the source and ***X****_θ_*_,_*_φ_*_,_*_γ_*_,_*_η_* (*t*) ∈ C^6×^*^N^*.

In the presence of *M* impinging signals and additive noise, the EMVS array output is given by:

(4) $$X(t)=\sum_{m=0}^{M}X_{\theta_{m},\varphi_{m},\gamma_{m},\eta_{m}}(t)+N(t)=\sum_{m=0}^{M}p_{m}d_{m}^{T}s_{m}(t)+N(t)$$

where (*θ_m_*,*φ_m_*), (*γ_m_*,*η_m_*) are the 2D angular and polarization parameters of the *mth* signal *s_m_*(*t*), ***p****_m_* ≜ ***p***_*θ_m_, φ_m_, γ_m_, η_m_*_, ***d****_m_* ≜ ***d***_*θ_m_, φ_m_*_, and *N*(*t*) ∈ C^6×*N*^ is the noise term, *m* =1, 2, …, *M*.

In addition, we herein give the following assumptions:

1. The sources are zero-mean, stationary, and mutually uncorrelated;
2. The noises are zero-mean, stationary, and uncorrelated with the sources.
3. The sources have distinct DOA’s, and any arbitrary *K* (*K* ≤ *N*) spatial steering vectors associated with different DOA’s are linearly independent.

Next, we shall show how to formulize the second-order statistics of the EMVS array output given in Equation (4) into a set of jointly diagonalizable square matrices.

### 2.2. Formulation of the Second-Order Statistics into Jointly Diagonalizable Matrices

We denote the outputs of the *nth* EMVS’s as ***x****_n_*(*t*) ∈ C^6^. By definition, we know that ***x****_n_*(*t*) is the *nth* column of ***X***(*t*) and thus could be further formulized as $x_{n}(t)=\sum_{m=1}^{M}p_{m}d_{m,n}s_{m}(t)+n_{n}(t)$, where *d*_*m,n*_ is the *nth* element of ***d****_m_*, and ***n****_n_*(*t*) ∈ C^6^ is the noise term on this EMVS. As a result, performing cross covariance between ***x****_l_*(*t*) and ***x****_k_*(*t*) under assumptions 1 and 2 yields (*l*, *k* =1, 2, …, *N*):

(5) $$C_{l,k}≜E[x_{l}(t)x_{k}^{H}(t)]={\mathrm{PR}}_{l,k}P^{H}+\Sigma_{l,k}$$

where ‘E(·)’ denotes mathematical expectation, ***P*** ≜ [***p***_1_, ***p***_2_,…, ***p****_M_*], ***R****_l,k_* ≜ diag(*r*_1,_*_l_*_,_*_k_*, *r*_2,_*_l_*_,_*_k_*,…, *r_M_*_,_*_l_*_,_*_k_*) where diag(·) construct a diagonal matrix with its entries, and $r_{m,l,k}≜E[s_{m}(t)s_{m}^{*}(t)]d_{m,l}d_{m,k}^{*}$. In addition, $\Sigma_{l,k}≜E[n_{l}(t)n_{k}^{H}(t)]$ is the noise cross-covariance matrix between the *lth* and *kth* EMVS’s. We note herein that for *l*, *k* varying between 1 and *N* there are totally *N*^2^ such matrices. Moreover, these matrices all admit Equation (5), which indicates a featured structure that distinct diagonal matrices {*R_l_*_,_*_k_* |*l*, *k* ∈ [1, *N*]} are multiplied by jointly shared target matrices ***P*** and ***P****^H^* from the left and right (in the absence of noise), respectively. This particular structure is called the jointly diagonalizable structure, and a unique identification of it would yield estimates of DOA and polarizations (as will be addressed in Section 3). In the open literature, joint diagonalization has been widely investigated with various constraints (such as Hermitianity and orthogonality) [45–57] for different applications. As such, we have the following remarks concerning the jointly diagonalizable structure present in our problem, as a guidance for the design of the CNJD algorithms.

*Remark 1*: It is important to note that the target matrix ***P*** is complex-valued and non-orthogonal as $p_{\theta_{1},\varphi_{1},\gamma_{1},\eta_{1}}^{H}p_{\theta_{2},\varphi_{2},\gamma_{2},\eta_{2}}\neq 0$ in most of the cases. Moreover, ***C****_l_*_,_*_k_* is non-Hermitian for *l* ≠ *k*. As a result, it is preferable to use complex non-orthogonl joint diagonalization (CNJD) methods for non-Hermitian matrices, for the identification of ***P***.

*Remark 2*: We note that most of the joint diagonalization algorithms assume that the target matrix be square, while in our problem ***P*** is mostly not square. As a result, we modify Equation (5) a little bit as follows (here we assume *M* ≤ 6):

(6) $$C_{l,k}=\underset{A\in C^{6\times 6}}{\underset{︸}{\left[P,P^{\prime }\right]}}\underset{D_{l,k}\in C^{6\times 6}}{\underset{︸}{\left[\begin{array}{cc} R_{l,k} & \\ & R_{l,k}^{\prime } \end{array}\right]}}\underset{A^{H}\in C^{6\times 6}}{\underset{︸}{{\left[P,P^{\prime }\right]}^{H}}}+\underset{\Sigma_{l,k}^{\prime }\in C^{6\times 6}}{\underset{︸}{\left(\Sigma_{l,k}-P^{\prime }R_{l,k}^{\prime }{P^{\prime }}^{H}\right)}}$$

where ***R***′*_l,k_* ∈ C^(6−*M*)×(6−*M*)^ is diagonal, ***P***′ ∈ C^6×(6−*M*)^, and ***P***′***R***′_*l,k*_***P***′*^H^* could be considered as the contribution from the noise term to the jointly diagonalizable structure. As a result, we will consider the model given in Equation (6) instead of Equation (5) for our proposed algorithms in the next section.

## 3. Proposed Algorithm

As indicated in Section 2, the EMVS array signals could be formulated in a set of complex non-orthogonal jointly diagonalizable matrices in the domain of second-order statistics, and the problem of joint DOA and polarization estimation might be solved by identifying this structure via CNJD algorithms. In this section, we firstly propose two CNJD algorithms using the Jacobi-type scheme and LU/LQ decompositions. Then we propose a novel strategy for simultaneous direction finding and polarization estimation based the proposed CNJD algorithms, as well as some discussion.

### 3.1. Complex Non-Orthogonal Joint Diagonalization via Jacobi-Type Scheme and LU/LQ Decompositions

We assume a set of complex matrices ***C*** = {***C***_1_, …, ***C****_K_*} share the jointly diagonalizable structure as given in Equation (6): ***C**_k_* = ***AD**_k_**A**^H^*. Here we neglect the noise term and combine the two indices (*l*,*k*) for *C_l,k_* and ***D**_l,k_* in Equation (6) as one index *k* for clarity. Moreover, we consider the general case that {***C***_1_, …, ***C****_K_*} are of size *L* × *L* for the proposed CNJD algorithms (in our particular case of joint DOA and polarization estimation with EMVS’s, *L* is equal to 6 according to Equation (6)). The goal of joint diagonalization is to seek an estimate for ***B*** ≜ ***A***^−^^1^ such that {***BC_k_B**^H^*} are as diagonal as possible. To solve this problem, we consider the following criterion for the estimation of ***B***:

(7) $$\tilde{B}=\text{arg}\;\underset{B}{\text{min}}\sum_{k=1}^{K}\text{off}(BC_{k}B^{H})$$

where off(***BC**_k_**B**^H^*) calculates the sum of squared norms for all the off-diagonal elements of ***BC**_k_**B**^H^*. In addition, by reasonably assuming that ***B*** is with unit determinant, we could further decompose it as:

(8) B=LV

where ***L*** ∈ C^*L×L*^ is lower-triangular with ones at its diagonal. ***V*** ∈ C*^L^*^×^*^L^* is upper-triangular if Equation (8) denotes LU decomposition, and is unitary if Equation (8) denotes LQ decomposition. Noting that any non-singular square matrix admits these two decompositions, it is reasonable to consider the decompositions Equation (8) and hence replace the minimization problem of Equation (7) by two alternating stages involving the following sub-optimization steps:

(9a) $$\tilde{V}=\text{arg}\;\underset{V}{\text{min}}\sum_{k=1}^{K}\text{off}({\mathrm{VC}}_{k}V^{H})$$

(9b) $$\tilde{L}=\text{arg}\;\underset{L}{\text{min}}\sum_{k=1}^{K}\text{off}({\mathrm{LC}}_{k}^{\prime }L^{H})$$

where ***C***′*_k_*, = ***ṼC****_k_****Ṽ****^H^*, ***L̃***, ***Ṽ***, and ***B̃*** denote the estimates of ***L***, ***V***, and ***B*** respectively.

Moreover, we adopt the Jacobi-type scheme to solve Equations (9a) and (9b) via a set of rotations which are repeated sequentially for all the possible index pairs (*i*, *j*) as follows, 1 ≤ *i* < *j* ≤ *L*:

(10a) $$C_{k,\mathrm{new}}=T_{\left(i,j\right)}C_{k,\mathrm{old}}T_{\left(i,j\right)}^{H},\;V_{\mathrm{new}}=T_{\left(i,j\right)}V_{\mathrm{old}}$$

(10b) $$C_{k,\mathrm{new}}^{\prime }=T_{\left(i,j\right)}^{\prime }C_{k,\mathrm{old}}^{\prime }T_{\left(i,j\right)}^{\prime H},\;L_{\mathrm{new}}=T_{\left(i,j\right)}^{\prime }L_{\mathrm{old}}$$

where ***C****_k_*_,_*_new_*, ***C***′*_k_*_,_*_new_*, ***V****_new_* and ***L****_new_* denote the updates of ***C****_k_*, ***C****′_k_*, ***V*** and ***L*** in the current iteration, and ***C****_k_*_,_*_old_*, ***C****′_k_*_,_*_old_*, ***V****_old_* and ***L****_old_* are the results obtained in the previous iteration, *k* = 1, …, *K*. ***T***_(_*_i_*_,_*_j_*_)_ and ***T****′*_(_*_i_*_,_*_j_*_)_ are the elementary rotation matrices for problems Equations (9a) and (9b), respectively, which equal the identity matrix except the entries indexed (*i*, *i*), (*i*, *j*), (*j*, *i*), and (*j*, *j*). The goal is then to find optimal ***T***_(_*_i_*_,_*_j_*_)_ and ***T***′_(_*_i_*_,_*_j_*_)_ in each iteration to solve Equations (10a) and (10b), respectively. We herein note that the proposed two CNJD algorithms are all of the above-mentioned Jacobi-type, with the only differences on the adopted decompositions (LU or LQ) and implementation details. Next, we give the details of the proposed algorithms.

Firstly, we consider the LU decomposition based algorithm. In this case, the matrix ***V*** in Equation (9a) is an upper-triangular matrix *U* ∈ C*^L^*^×^*^L^*, and ***T***_(_*_i_*_,_*_j_*_)_ in Equation (10b) is an elementary upper-triangular matrix which is equal to the identity matrix except the (*i*, *j*)*th* upper entry *α_i,j_*, 1 ≤ *i* < *j* ≤ *L*. In addition, for index pair (*i*, *j*), we note that $T_{\left(i,j\right)}C_{k,\mathrm{old}}T_{\left(i,j\right)}^{H}$ only impacts the *ith* row and column of ***C****_k_*_,_*_old_*, *k* = 1, …, *K*, and thus the minimization of $\sum_{k=1}^{K}\text{off}(T_{\left(i,j\right)}^{H}C_{k,\mathrm{old}}T_{\left(i,j\right)})$ amounts to minimizing the sum-of squared norms of the off-diagonal elements in the *ith* row and column of ***C****_k_*_,_*_new_*:

(11) $$\xi_{i,j}≜\sum_{k=1}^{K}\sum_{p=1,p\neq i}^{6}\left[{\left|C_{k,\mathrm{new}}(i,p)\right|}^{2}+{\left|C_{k,\mathrm{new}}(p,i)\right|}^{2}\right]$$

We note that *C_k_*_,_*_new_* (*i*, *p*) = *α_i_*_,_*_j_* *C_k_*_,_*_old_* (*j*, *p*) + *C_k_*_,_*_old_* (*i*, *p*) and $C_{k,\mathrm{new}}(p,i)=C_{k,\mathrm{old}}(p,i)+\alpha_{i,j}^{*}C_{k,\mathrm{old}}(p,j)$. As a result, substituting these formulations into Equation (11) yields the following equation after a few simple manipulations:

(12) $$\begin{array}{lll} \xi_{i,j} & = & \sum_{k=1}^{K}\sum_{p=1,p\neq i}^{L}\{(C_{k,\mathrm{old}}\;(j,p)C_{k,\mathrm{old}}^{*}\;(j,p)+C_{k,\mathrm{old}}\;(p,j)C_{k,\mathrm{old}}^{*}\;(p,j))\alpha_{i,j}\;\alpha_{i,j}^{*}\\ & & +[C_{k,\mathrm{old}}\;(i,p)C_{k.\mathrm{old}}^{*}\;(j,p)+C_{k.\mathrm{old}}\;(p,j)C_{k.\mathrm{old}}^{*}\;(p,i)]\alpha_{i,j}^{*}\\ & & +[C_{k.\mathrm{old}}\;(j,p)C_{k,\mathrm{old}}^{*}\;(i,p)+C_{k.\mathrm{old}}\;(p,i)C_{k.\mathrm{old}}^{*}\;(p,j)]\alpha_{i,j}\\ & & +[C_{k.\mathrm{old}}\;(i,p)C_{k.\mathrm{old}}^{*}\;(i,p)+C_{k.\mathrm{old}}\;(p,i)C_{k,\mathrm{old}}^{*}\;(p,i)]\} \end{array}$$

Setting the derivative of *ξ_i,j_* with regards to $\alpha_{i,j}^{*}$ yields the optimal estimate of *α_i,j_* as:

(13) $${\tilde{\alpha }}_{i,j}=-\frac{\sum_{k=1}^{K}\sum_{p=1,p\neq i}^{L}\left[C_{k,\mathrm{old}}\;(i,p)C_{k,\mathrm{old}}^{*}\;(j,p)+C_{k,\mathrm{old}}\;(p,j)C_{k,\mathrm{old}}^{*}\;(p,i)\right]}{\sum_{k=1}^{K}\sum_{p=1,p\neq i}^{L}\left[C_{k,\mathrm{old}}\;(j,p)C_{k,\mathrm{old}}^{*}\;(j,p)+C_{k,\mathrm{old}}\;(p,j)C_{k,\mathrm{old}}^{*}\;(p,j)\right]}$$

As for the L-stage, we note herein that the only difference is the use of lower-triangular matrix instead of the upper-triangular one in the U-stage. Therefore, Equation (13) is still valid for the optimal estimate of the factor involved in the L-stage, except that the indices *i* and *j* are transposed.

In the second algorithm, we consider the LQ decomposition of ***B***, and thus the matrix ***V*** in Equation (9a) is a unitary matrix ***Q*** ∈ C*^L^*^×^*^L^*. Hence, the sub-optimization problem in the Q-stage as indicated in Equation (9a) is indeed an orthogonal joint diagonalization (OJD) problem which could be solved by Cardoso’s Jacobi-type algorithm [45]. As such, in the second algorithm we use Cardoso’s OJD algorithm in the Q-stage, followed by the L-stage which is addressed in the first proposed algorithm.

Now that we have obtained the L-stage and U(Q)-stage for the proposed algorithms, we loop these two stages until convergence is reached. In addition, we note that there would be several ways to monitor the convergence for the proposed CNJD algorithms. For example, we could terminate the iterations when the parameter values in each iteration of the L-stage or U(Q)-stage are small enough, which indicate a minute contribution from the elementary rotations, and hence convergence. We might as well monitor the sum of off-diagonal squared norms $\sum_{k=1}^{K}\text{off}({\mathrm{BC}}_{k}B^{H})$ in each iteration, and terminate the loops when the change in it is smaller than a preset threshold. In the following context of our manuscript, we adopt a third way which stops the iterations when the values of ${\left\|\mathrm{LV}-I\right\|}_{F}^{2}$ between two successive complete runs of L-stage and U(Q)-stage are close enough, with regards to a preset threshold τ, where ***I*** denotes the identity matrix. We name the proposed LU and LQ based algorithms as LUCJD and LQCJD, respectively, and summarize them in Table 1.

### 3.2. Joint DOA and Polarization Estimation with CNJD

Given the proposed LUCJD and LQCJD algorithms, we could identify the jointly diagonalizable structure of an EMVS array outputs as given in Equation (6), for the estimation of ***A*** = ***B***^−1^. Moreover, we note that the identification of ***A*** implies estimates of the angular-polarization steering vectors ***p****_m_*, which could be used to estimate the source DOA’s and polarizations. In addition, it is important to note that the target matrices as given in Equation (5) are all of dimensionality 6 × 6, and therefore the sensitivity issue for non-orthogonal JD with very few large matrices would not occur in our applications.

However, it is important to note that ***A*** is required to be square for the proposed CNJD algorithms, and thus the estimation of ***A*** by using these algorithms does not only contain the desired estimates of ***p****_m_*, but also the contribution from the noise, as is formulated in Equation (6) (we denote the estimates of ***A***, ***B***, and ***p****_m_* by ***Ã***, ***B̃***, and ***p̃****_m_*, respectively, *m* =1, 2, …, *M*). Therefore, before performing joint DOA and polarization estimation, we need to firstly separate the columns of ***Ã*** associated with the sources from those contributed by noise. This procedure is similar to signal/noise subspace estimation which is key to subspace based methods such as MUSIC and ESPRIT. However, noting that ***Ã*** is not constrained to be orthogonal, we could not separate its columns into the signal and noise subspaces by simply selecting the columns corresponding to the largest *M* diagonal elements of {***B̃C****_l,k_* ***B̃****^H^*}, as is done for MUSIC and ESPRIT. As such, we propose the following scheme to solve this problem.

Firstly, we stack all the matrices ***C****_l,k_* as given in Equation (5) into a big matrix ***C*** ∈ C^36×*N*^2^^ as :

(14) $$C=[\text{vec}(C_{1,1}),\text{vec}(C_{2,1}),...\text{vec}(C_{N,1}),...,\text{vec}(C_{1,N}),\text{vec}(C_{2,N}),...,\text{vec}(C_{N,N})]$$

where ‘vec(·)’ denotes matrix vectorization. Then we perform singular value decomposition (SVD) to ***C*** ∈ C^36×*N*^2^^, denote the number of singular values as *R* = min(36, *N*^2^), and select the right SVD vectors associated with the smallest *R* − *M* singular values, denoted by ***U*** ≜ [***u****_M_*_+1_, ***u****_M_*_+2_,…, ***u****_R_*] ∈ C*^N^2^×(R−M)^*, to estimate the noise subspace which is orthogonal to the span of $\left\{d_{1}⊗d_{1}^{*},\ldots ,d_{M}⊗d_{M}^{*}\right\}$, where ***d****_m_* is the spatial steering vector of the *mth* source, as given in Equation (2). In addition, we construct a noise subspace projector ***Π*** ≜ ***UU**^H^*. We note herein that the computational complexity of this stage would grow dramatically as the number of sensors increases.

Secondly, we note that ***B̃C****_l,k_* ***B̃****^H^* is actually an estimate of ***D****_l_*_,_*_k_* in Equation (6), and $\mu_{l,k}≜\text{diag}(D_{l,k})={\left[\Xi {\left[d_{1,l}d_{1,k}^{*},...,d_{M,l}d_{M,k}^{*}\right]}^{T},{\left(\text{diag}(R_{l,k}^{\prime })\right)}^{T}\right]}^{T}$ where ***Ξ*** ∈ C^*M×M*^ is a diagonal matrix with the *mth* diagonal element equal to $E[s_{m}(t)s_{m}^{*}(t)]$, according to Equations (5) and (6), *l*,*k* = 1, …, *N*. As a result, we have:

(15)

which indicates that the columns of *Γ* ∈ C^*N*^2^×6^ comprise the vectors $\left\{d_{m}⊗d_{m}^{*}\right\}$ scaled by *Ξ*, and some noise contribution denoted as [diag(***R***′_1,1_)], diag(***R***′_1,2_),..., diag(***R***′*_N_*_,_*_N_*)]*^T^*. As a result, projecting these columns into the noise subspace via the projector ***Π*** could yield an efficient separation of the column space for ***Γ*** into signal and noise subspaces. Therefore, we perform the following projection for each column of ***Γ*** and select the column indices corresponding to the signal subspace:

(16) $$\text{index}=\underset{i}{\text{arg min norm}}(\Pi \Gamma (:,i))$$

where we adopt matlab notation to denote the *ith* column of ***Γ*** by ***Γ***(:,*i*). We note that there are totally *M* such indices which could be put into an index vector *λ* ∈ R*^M^*.

Finaly, we use the indices obtained above to select the columns of ***Ã*** that correspond to the estimates of the angular-polarization steering vectors ***p̃****_m_* = ***Ã***(;,*λ_m_*).

With the obtained estimates {*p̃_m_* | *m* = 1,2,...,*M*}, and denoting ${\left[{\tilde{e}}_{m}^{T},{\tilde{h}}_{m}^{T}\right]}^{T}={\tilde{p}}_{m}$, where ***ẽ****_m_*, ***h̃****_m_* ∈ C^3^, we adopt the scheme in [13,14] to calculate the DOA and polarization estimates as follows:

(17) $$\left\{ \begin{array}{l} v_{\left({\tilde{\theta }}_{m},{\tilde{\varphi }}_{m}\right)}={\left[\text{cos}\;{\tilde{\theta }}_{m}\;\text{cos}\;{\tilde{\varphi }}_{m},\;\text{sin}\;{\tilde{\theta }}_{m}\;\text{cos}\;{\tilde{\varphi }}_{m},\;\text{sin}\;{\tilde{\varphi }}_{m}\right]}^{T}={\tilde{e}}_{m}\times {\tilde{h}}_{m}^{*}\\ {\tilde{\gamma }}_{m}=\text{arctan}(|{\overset{⌢}{a}}_{m}(3)/{\overset{⌢}{a}}_{m}(6)|);\;{\tilde{\eta }}_{m}=\text{arg}(-\vec{i}\;{\overset{⌢}{a}}_{m}(3)/{\overset{⌢}{a}}_{m}(6)) \end{array}\right.$$

We summarize the proposed joint DOA and polarization estimation strategy in Table 2.

### 3.3. Discussion

In this subsection we present some remarks to provide insights into the proposed CNJD algorithms and the joint DOA and polarization estimator:

*Remark 3 (Concerning the adopted CNJD algorithms)*: Besides the proposed LUCJD and LQCJD methods, we could also use other joint diagonalization algorithms for the considered problem of simultaneous DOA and polarization estimation. We herein note that our proposed algorithms are of nice flexibility in various applications, as they assume neither Hermitianity nor positive definiteness on the matrices, which are often imposed by other CNJD algorithms [46,47,57]. In addition, compared with CNJD methods based on gradient or Newton-type optimization [51,52], the proposed algorithms are of closed-form in each iteration, and do not involve issues such as learning rate adjustment. Furthermore, we note that our algorithms could be considered as a generalization of the works in [53–55], which considered parametrized Jacobi-type non-orthogonal joint diagonalization for real-valued matrices. In particular, the work in [53] firstly considered non-orthogonal Jacobi-like algorithms based on LU and LQ decompositions, and was extended to the complex domain in [57]. However, we note that the algorithm in [57] considered only Hermitian matrices, and is thus essentially distinct from our proposed algorithms. There are also some other Jacobi-like algorithms, most of which consider only the real-valued cases [53–56]. Recently, there have been works devoting to Jacobi-like complex non-orthogonal CNJD algorithms [50,57].

*Remark 4(On the link to tensorial methods)*: It should be noted that our problem could also be tackled by tensorial methods. In particular, stacking the matrices {***C****_l_*_,_*_k_* | *l*,*k* = 1, …, *N*} given in Equation (5) along a third dimension would yield a trilinear tensor [35,36], and these matrices could also be arranged into a quadralinear tensor according to [37]. Therefore, the joint estimates for DOA and polarization could be obtained by performing parallel factor analysis (PARAFAC) upon these tensor models. Compared with these tensorial methods, we note that the CNJD mostly considered the ‘square’ case as indicated in *Remark 2* while PARAFAC could handle the non-square case. As a result, the CNJD based strategy is usually combined with subspace methods to select the desired columns from the square estimates.

Another noteworthy property of CNJD based strategies is that CNJD is usually very robust to noise with structured covariance, which would yield additional contribution to the joint diagonalizable structures other than sources. Therefore, the proposed CNJD based strategy might be robust to colored noise or interferences. On the other hand, the mostly used alternating least squares (ALS) methods for the implementation of PARAFAC are sometimes sensitive to erroneous prediction of the underlying parallel factors (e.g., underfactoring) [58], such that the presence of coherent noise with structured covariance would deteriorate the performance of PARAFAC based strategies, even if the number of sources is correctly predicted. However, in contrary to the above nice property, the CNJD algorithms are sensitive to unstructured noise such as white noise. In practice, we note that the array noise is usually spatially colored with the covariance between different elements admitting some particular structure [44], and therefore the CNJD based strategy would be well adapted to practical scenarioes.

*Remark 5(On the independence of knowledge on spatial configuration)*: A main property of the proposed method is that the joint estimation for DOA and polarization mainly relies on the estimates of angular-polarization steering vectors, while the estimates of spatial steering vectors that are indicated in the diagonal elements of {***B̃C****_l,k_****B̃****^H^*} are only used in the noise subspace projection stage to select the desired columns of the estimate ***Ã***. As such, the proposed approach does not require any knowledge on the spatial configuration of the array, and is therefore of much flexibility in applications where precise knowledge on the position of each EMVS is unavailable. Meanwhile, the above independence of the spatial prior knowledge for the proposed strategy would lead to another advantage, that the adopted array might be spatially sparse, which enables enlarged spatial aperture.

*Remark 6(On the identifiability)*: An issue of great interests is the identifiability property of the proposed strategy and this issue comprises two aspects, that are: (1) the least number of EMVS’s that are required for a successful implementation of the proposed method; and (2) the most number of sources that could be uniquely identified. To address the former, we note that the jointly diagonalizable structure could be uniquely identified for at least two matrices, and hence we need at least two EMVS’s for the proposed method (a simple example is the ESPRIT algorithm). For the latter issue, we simply notice that the number of sources could not exceed six to meet the “square” requirement of the proposed LUCJD and LQCJD algorithms. We note herein that the above analysis is given under assumption A3) in Section II, which excludes the scenarioes that two or more target matrices for CNJD are proportional or linearly dependent. A more challenging problem is the identification of several sources with identical DOA, and this requires a thorough analysis of the identifiability for CNJD. However, this issue is beyond the scope of this manuscript and will be the focus of our future work.

*Remark 7(On the computational complexity)*: Another important issue about the proposed LUCJD and LQCJD is the computational complexity, and this issue comprises two aspects, that are: (1) The computational complexity for one sweep; and (2) the number of sweeps until convengence. For the former, we note that one sweep in these algorithms comprises two stages, each of which undergoes *L*(*L* – 1)/2 elementary rotations (*L* is the dimensionality of the target matrices). Moreover, we note that the update of target matrices involve 2*L*^2^ complex multiplication operations and the update of matrices ***L*** or ***U*** (***Q***) involve *L*^2^ complex multiplications according to Equation (10). The calculation of the optimal *α_i_*_,_ *_j_* involves 4*K*(*L* – 1) multiplications (there is also a division, ignored as it contributes little to the complexity) according to Equation (3). The Q-stage in LQCJD involves 9*K* multiplications and an eigendecomposition of a 3 × 3 matrix. Therefore, the computational loads of one sweep of LUCJD and LQJCD are 3*L*^3^(*L* – 1) + 4*KL*(*L* – 1)^2^, and 3*L*^3^(*L* – 1) + 2*KL*(*L* – 1)^2^ + (9*K* + *ξ*)*L*(*L* – 1)/2, respectively, where *ξ* is the complexity of computing the eigendecomposition of a 3 × 3 matrix. We note herein that the computational complexity of LUCJD and LQCJD per sweep is approximately larger than that of other Jacobi-type algorithms (e.g., JTJD [50]) as each sweep of LUCJD and LQCJD incorporates two stages while other methods only have one stage in each sweep.

As for the number of sweeps that LUCJD and LQCJD take until convergence, we note that LQCJD converges nearly quadratically, mainly because the algorithm in the Q-stage is of quadratic converging pattern. The converging pattern of LUCJD, however, is linear as inherited from its real counterpart [53].

## 4. Simulation Results

In this section, we present simulations to verify the proposed joint DOA and polarization estimation strategy, and compare it with other methods of similar type (e.g., tensorial algorithms). The impinging signals are assumed to be far-field narrow-band signals of equal carrying frequency, and the adopted array is a sparse linear array with interspacing identical to twice the wavelength (the number of sources and EMVS’s are set differently in various simulations). In addition, the noise is formulated following the reports on sensor array noise modeling [44] as:

(18a) $$E(n_{e,i,k}n_{e,j,k^{\prime }}^{*})=\{\begin{array}{ll} p_{\varepsilon }\;\rho_{1}^{\left|i-j\right|}d_{\theta_{\varepsilon },\varphi_{\varepsilon },i}\;d_{\theta_{\varepsilon },\varphi_{\varepsilon },j}^{*} & k=k^{\prime }\\ p_{\varepsilon }\;\rho_{1}^{\left|i-j\right|}\rho_{2}\;d_{\theta_{\varepsilon },\varphi_{\varepsilon },i}\;d_{\theta_{\varepsilon },\varphi_{\varepsilon },j}^{*}e_{\theta_{\varepsilon },\varphi_{\varepsilon },\gamma_{\varepsilon },\eta_{\varepsilon },i}\;e_{\theta_{\varepsilon },\varphi_{\varepsilon },\gamma_{\varepsilon },\eta_{\varepsilon },j}^{*} & k\neq k^{\prime } \end{array}$$

(18b) $$E(n_{h,i,k}\;n_{h,j,k^{\prime }}^{*})=\{\begin{array}{ll} p_{\varepsilon }\;\rho_{1}^{\left|i-j\right|}d_{\theta_{\varepsilon },\varphi_{\varepsilon },i}\;d_{\theta_{\varepsilon },\varphi_{\varepsilon },j}^{*} & k=k^{\prime }\\ p_{\varepsilon }\;\rho_{1}^{\left|i-j\right|}\rho_{2}\;d_{\theta_{\varepsilon },\varphi_{\varepsilon },i}\;d_{\theta_{\varepsilon },\varphi_{\varepsilon },j}^{*}h_{\theta_{\varepsilon },\varphi_{\varepsilon },\gamma_{\varepsilon },\eta_{\varepsilon },i}\;h_{\theta_{\varepsilon },\varphi_{\varepsilon },\gamma_{\varepsilon },\eta_{\varepsilon },j}^{*} & k\neq k^{\prime } \end{array}$$

(18c) $$E(n_{e,i,k}\;n_{h,j,k^{\prime }}^{*})=\{\begin{array}{ll} p_{\varepsilon }\;\rho_{1}^{\left|i-j\right|}d_{\theta_{\varepsilon },\varphi_{\varepsilon },i}\;d_{\theta_{\varepsilon },\varphi_{\varepsilon },j}^{*} & k=k^{\prime }\\ p_{\varepsilon }\;\rho_{1}^{\left|i-j\right|}\rho_{2}\;d_{\theta_{\varepsilon },\varphi_{\varepsilon },i}\;d_{\theta_{\varepsilon },\varphi_{\varepsilon },j}^{*}e_{\theta_{\varepsilon },\varphi_{\varepsilon },\gamma_{\varepsilon },\eta_{\varepsilon },i}\;h_{\theta_{\varepsilon },\varphi_{\varepsilon },\gamma_{\varepsilon },\eta_{\varepsilon },j}^{*} & k\neq k^{\prime } \end{array}$$

where *p_ε_* is the noise power, 0 ≤ *ρ*_1_ ≤ 1 is the noise covariance level between parallel components of two neighboring EMVS’s, and 0 ≤ *ρ*_2_ ≤ 1 is the noise covariance level between two orthogonal components within the same EMVS. *d_θ_*_*_ε_*__,_*_φ_*_*_ε_*__,_*_i_*, *e_θ_*_*_ε_*__,_*_φ_*_*_ε_*__,_*_γ_*_*_ε_*__,_*_η_*_*_ε_*__,_*_i_*, and *h_θ_*_*_ε_*__,_*_φ_*_*_ε_*__,_*_γ_*_*_ε_*__,_*_η_*_*_ε_*__,_*_i_* denote the *ith* elements of *d_θ_*_*_ε_*__,_*_φ_*_*_ε_*_, *e_θ_*_*_ε_*__,_*_φ_*_*_ε_*__,_*_γ_*_*_ε_*__,_*_η_*_*_ε_*_, and ***h**_θ_*_*_ε_*__,_*_φ_*_*_ε_*__,_*_γ_*_*_ε_*__,_*_η_*_*_ε_*_, respectively, as defined in Equations (1) and (2), with (*θ_ε_*, *φ_ε_*) and (*γ_ε_*, *η_ε_*) denoting noise DOA and polarization, respectively. ***n****_e_*_,_*_i_*_,_*_k_*, ***n****_h_*_,_*_i_*_,_*_k_* denote the noise on the *kth* short dipole and small loop in the *ith* EMVS, respectively. We note that this formulation takes into consideration the features of both white noise and directional interference. In particular, this noise model degrades to the white noise model with *ρ*_1_ = *ρ*_2_ = 0, and becomes an interference with DOA (*θ_ε_*, *φ_ε_*) and polarization (*γ_ε_*, *η_ε_*) with *ρ*_1_ = *ρ*_2_ = 1. Furthermore, in our simulations we fix the noise DOA (*θ_ε_, φ_ε_*) = (47°, 45°), and polarization (*γ_ε_*, *η_ε_*) = (0°, 0°), respectively.

We use the Overall *Root Mean Squared Angular Error* (RMSAE) [2] to measure the accuracy of DOA estimates as $\chi ≜M^{-1}\sum_{m=1}^{M}\sqrt{E(\text{arccos}(v_{\theta_{m},\varphi_{m}}^{T}v_{{\tilde{\theta }}_{m}\;{\tilde{\varphi }}_{m}}))}$, where *v_θ_*_*_m_*__,_*_φ_*_*_m_*_ and *v_θ̃_*_*_m_*__,_*_φ̃_*_*_m_*_ are the *mth* true and estimated poynting vectors, respectively. In addition, we use the Overall *Mean Squared Errors* (RMSE) to measure the estimation accuracy of the two polarization parameters. Furthermore, SNR used in all the simulations is defined as *SNR* ≜ 10log_10_ (*p_s_* / *p_ε_*), where *p_s_* and *p_ε_* are the signal power and noise power per vector-sensor, respectively. In the following, the first simulation is performed to verify our identification analysis in *Remark 6*, Section 3. In the second simulation, the proposed strategy is compared with other methods of similar type, with regards to the robustness to different errors (e.g., the errors caused by noise, finite data length, and noise covariance). The third simulation concerns the converging pattern of the proposed LUCJD and LQCJD algorithms.

### 4.1. Simulation for Six Sources Incident on Two EMVS’s

**Figure 3.**
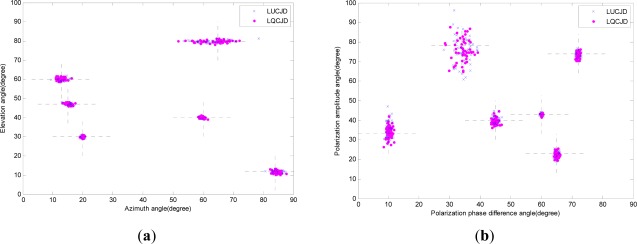
The distribution of DOA and polarization estimates from 50 independent runs, SNR is 30dB, the number of snapshots is 1,000. The noise is with covariance levels (*ρ*_1_, *ρ*_2_) = (0.5, 0.5). (**a**) Distribution of DOA estimates. (**b**) Distribution of polarization estimates.

In the first simulation, we consider the scenario that six sources are impinging on an array of two EMVS’s, to verify our identification analysis in *Remark 6*, Section 3. We note that in this case the number of sources reaches the upper-bound, while the EMVS number reaches the lower-bound according to *Remark 6*, and thus this is the most difficult case to the identifiability for the proposed strategy. The source DOA’s are (*θ*_1_, *φ*_1_) = (47°, 15°), (*θ*_2_, *φ*_2_) = (12°, 84°), (*θ*_3_, *φ*_3_) = (40°, 60°), (*θ*_4_, *φ*_4_) = (30°, 20°), (*θ*_5_, *φ*_5_) = (60°, 13°), and (*θ*_6_, *φ*_6_) = (80°, 65°). The source polarizations are (*γ*_1_, *η*_1_) = (72°, 74°), (*γ*_2_, *η*_2_) = (60°, 43°), (*γ*_3_, *η*_3_) = (10°, 33°), (*γ*_4_, *η*_4_) = (65°, 23°), (*γ*_5_, *η*_5_) = (45°, 40°), and (*γ*_6_, *η*_6_) = (34°, 78°). In addition, we fix the SNR to 30 dB, fix the number of snapshots to 1000, set the noise covariance levels in Equations (18a)∼(18c) as (*ρ*_1_, *ρ*_2_) = (0.5, 0.5), and perform 50 independent runs for both LUCJD and LQCJD based algorithms. We here note that SNR in this scenario is set to a high level so that the impact of noise on the result could be ignored, and thus enabling a clearer illustration of the identifiability property of the proposed strategy. The distributions of DOA and polarization estimates are plotted in Figure 3. We note from the figure that the results from 50 independent runs assemble around the true values of the source DOA’s and polarizations denoted by crossed dashed lines, and this verifies our identifiability analysis in *Remark 6* that our proposed strategy could successfully identify at most six sources with at least two EMVS’s.

### 4.2. Comparison with Other CNJD and PARAFAC Based Algorithms

In the second simulation, we compare the proposed methods with the trilinear PARAFAC based algorithm, a natural competitor to the proposed algorithms, noting that they have similar features with regards of the modeling and processing of the trilinear MD structure, such that the pros and cons of both methods could be fairly illustrated. The quadrilinear PARAFAC, however, is not included in the comparison, as it exploits fourth-order multilinearity of the present data statistics, and thus is not a natural competitor to CNJD and trilinear PARAFAC which concern third-order multilinearity. In addition, it is also of great interests to see how the proposed strategy would perform if the CNJD stage is carried out with other algorithms instead of the proposed LUCJD and LQCJD. Hence, we shall also include in the comparisons some other CNJD methods such as the nonparametric Jacobi transformation based joint diagonalization (JTJD) [50], the fast approximate joint diagonalization (FAJD) [48], and the uniformly weighted exhausive diagonalization by Gaussian iteration (UWEDGE) [49]. We select these methods mainly due to their similar nature to the proposed LUCJD and LQCJD algorithms (complex, non-orthogonal, and do not require Hermianity nor non-negative definiteness). In addition, the PARAFAC based method in our comparison uses the trilinear PARAFAC model in the subspace domain, as introduced in [32], and adopts the joint diagonalization strategy [59] along with compression based factor analysis [60] algorithm (COMFAC) to accelerate the trilinear decomposition. For the compared CNJD algorithms, LUCJD, LQCJD, and JTJD are initialized with identity matrix, while FAJD and UWEDGE adopt the coarse outputs of the orthogonal joint diagonalization as the initialization. The reason for the above different initializations is that these CNJD algorithms are of two different types (LUCJD, LQCJD, and JTJD are Jacobi-like, and FAJD, UWEDGE are with the weighted least squares criterion), and we strive to make the comparisons fair by letting these algorithms operate with their standard settings.

We consider the scenario that three sources are impinging upon an array of six EMVS’s. The array configuration is given at the begining of Section 4. The source DOA’s are (*θ*_1_, *φ*_1_) = (47°, 15°), (*θ*_2_, *φ*_2_) = (12°, 84°), and (*θ*_3_, *φ*_3_) = (40°, 60°). The source polarizations are (*γ*_1_, *η*_1_) = (72°, 74°), (*γ*_2_, *η*_2_) = (60°, 43°), and (*γ*_3_, *η*_3_) = (10°, 33°).

**Figure 4.**
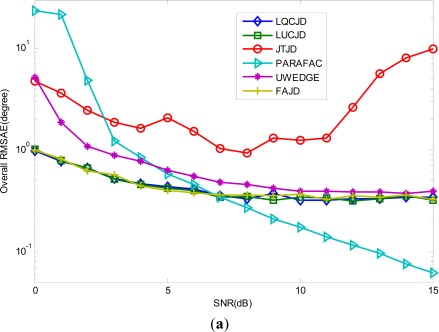

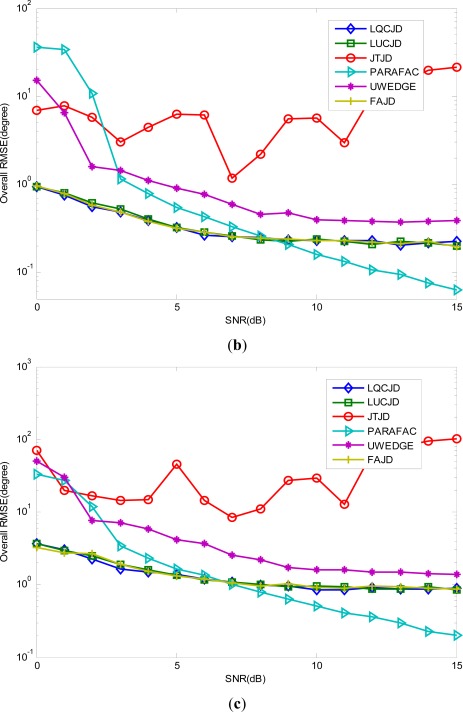
Performance of LUCJD, LQCJD, PARAFAC, UWEDGE, FAJD, JTJD *versus* SNR. The number of snapshots is 1,000, and the noise is with covariance levels (*ρ*_1_, *ρ*_2_) = (0.8, 0.8). (**a**) The overall RMSAE curves of DOA estimates. (**b**) The overall RMSE curves of polarization amplitude angle estimates. (**c**) The overall RMSE curves of polarization phase difference angle estimates.

Firstly, we set the noise covariance levels (*ρ*_1_, *ρ*_2_) = (0.8, 0.8), set the number of snapshots to 1,000, and let SNR vary between 0 dB∼15 dB. The results obtained from 100 independent runs are plotted in Figure 4. We observe from the results that the proposed LUCJD and LQCJD algorithms provide almost equal precision as well as FAJD, and UWEDGE slightly underperforms these three algorithms. Furthermore, we note that the curves of LUCJD, LQCJD, FAJD, and UWEDGE drop more smoothly than PARAFAC, which indicates an improved robustness to colored noise of CNJD based methods over the PARAFAC based one. In particular, the proposed LUCJD and LQCJD outperform PARAFAC for low SNR levels (0∼6 dB), while PARAFAC performs better for high SNR levels (7∼15 dB). In addition, we note that JTJD underperforms the other algorithms with regards to the overall accuracy of DOA and polarization estimates. The main reason is that JTJD behaves unsteadily in our simulations, and sometimes converges to false solutions (we could draw the distribution of DOA and polarization estimates similarly to Figure 3 for JTJD to clearly show that it converges to false solutions for quite a few independent runs). We also note that UWEDGE slightly underperforms FAJD, LUCJD, and LQCJD with regards to the estimation accuracy. This is mainly because the WLS criterion based algorithms usually perform better with a set of properly designed weights for target matrices while UWEDGE adopts identical ones only.

**Figure 5.**
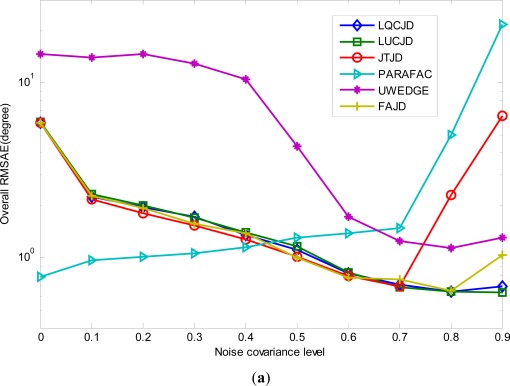

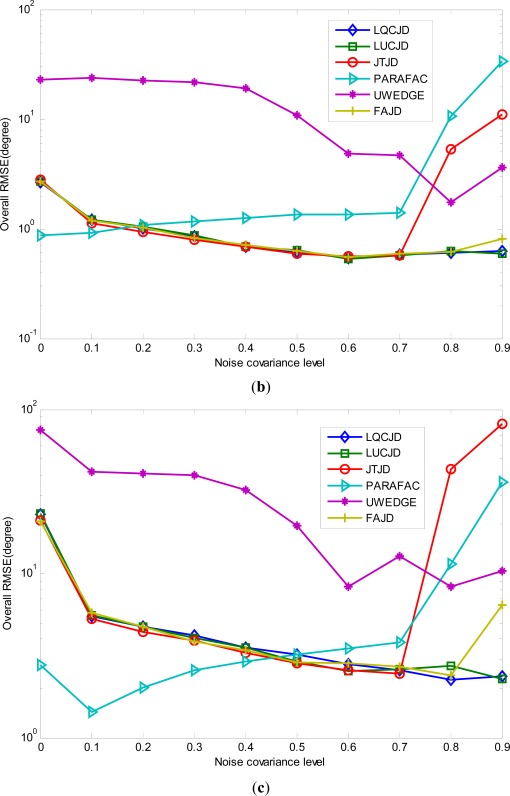
Performance of LUCJD, LQCJD, PARAFAC, UWEDGE, FAJD, JTJD *versus* the noise covariance level. SNR is 2 dB and the number of snapshots is 1,000. (**a**) The overall RMSAE curves of DOA estimates. (**b**) The overall RMSE curves of polarization amplitude angle estimates. (**c**) The overall RMSE curves of polarization phase difference angle estimates.

Secondly, we fix SNR and the number of snapshots to 2 dB and 1,000, respectively, and let the noise covariance levels *ρ*_1_ = *ρ*_2_ vary from 0∼0.9. The results from 100 independent runs are demonstrated in Figure 5. We can see quite diverse behaviors for CNJD and PARAFAC based strategies with the noise covariance changing, which clearly demonstrate the pros and cons of both strategies. More exactly, we note that the increase in noise covariance would generally improve the performance of CNJD based methods. In particular, the proposed LUCJD and LQCJD provide the best performance among all the CNJD algorithms with regards to the accuracy of joint DOA and polarization estimation. In addition, FAJD and JTJD provide almost equal performance as LUCJD and LQCJD for low noise covariance levels, yet become unsteady for *ρ*_1_ = *ρ*_2_ ≥ 0.8 and *ρ*_1_ = *ρ*_2_ ≥ 0.9, respectively. This is because the CNJD methods, especially the proposed LUCJD and LQCJD, are usually very robust to noise with structured covariance, which would yield additional contribution to the joint diagonalizable structures other than sources. Moreover, we note that PARAFAC, on the other hand, behaves inversely to the CNJD ones, as the increase in noise covariance results in a dramatic rise in the its curves. This is because the PARAFAC algorithms are usually sensitive to underfactoring (the number of parallel factors in the tensor model is greater than the number of sources) [58] and noise with high covariance levels would result in such underfactoring (when noise covariance increases, noise becomes interference, and thus contributes an additional factor to the PARAFAC model). The above observations are in accordance with our analysis in *Remark 4*, Section 3, that CNJD performs better than PARAFAC with regards to the robustness to color noise, yet the latter has stronger ability in suppressing white noise.

### 4.3. Comparison on the Convergence of CNJD Algorithms

In the third simulation, we examine the converging properties of all the compared CNJD algorithms via an iteration-by-iteration evaluation by the use of performance index (PI) [54] which is defined as:

(19) Index(Ω)=60−1[∑i=16(∑j=16|ωij|maxk|ωik|−1)+∑j=16(∑i=16|ωij|maxk|ωkj|−1)]

where ***Ω* = *B̃A***, of which the (*i*, *j*)*th* entry is denoted as ω*_ij_*, and max*_k_* denotes maximum along the index *k*. ***A*** is given in Equaiton (6) and *B****˜*** denotes its estimate at a certain iteration of CNJD.

In addition, we terminate the iterations for all the CNJD algorithms when the decrease in PI values for two successive iterations (normalized by the current PI value) is smaller than 10^−2^. We note that it is impossible to monitor the values of PI as is done in this simulation in practice, as the knowledge of ***A*** is generally unavailable. The reasons for us doing so are: (1) to emphasize the converging patterns of the compared algorithms which are best illustrated by PI values for all the undergone iterations; and (2) to enable a fairer comparison of these algorithms as they generally incorporate distinct stopping criteria in practice.

**Figure 6.**
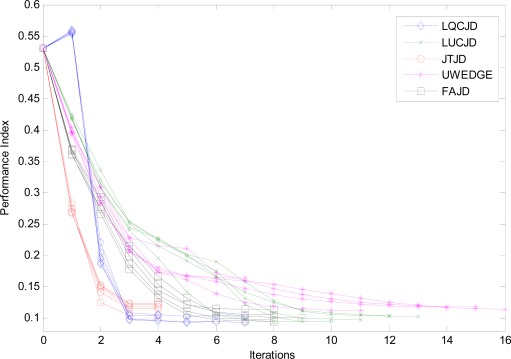
Performance indices of LUCJD, LQCJD, UWEDGE, FAJD, and JTJD *versus* iterations. SNR is 10 dB, the number of snapshots is 1,000, and the noise is with covariance levels (*ρ*_1_, *ρ*_2_) = (0.5, 0.5).

The simulation settings are the same as the first simulation except that SNR is set to 5 dB. We plot the PI curves from 5 independent runs for all the compared CNJD algorithms in Figure 6. We could observe that the proposed LQCJD yields similar nice quadratic converging pattern as JTJD, of which the PI curves drop dramatically in the fist 2∼3 iterations. The LUCJD algorithm, on the other hand, is less efficient than LQCJD, JTJD, and FAJD with regards to the number of iterations.

## 5. Conclusions

A new joint DOA and polarization estimation strategy was proposed with arrays of electromagnetic vector-sensors (EMVS). This method formulates the EMVS array signals into a set of matrices which admit a special jointly diagonalizable structure in the domain of second-order statistics, and further uses complex non-orthogonal joint diagonalization (CNJD) algorithms to identify this structure. Moreover, we project the columns of the identified target matrix into the estimated noise subspace, to distinguish the estimates of angular-polarization steering vectors from the noise turbulances, and further use them to obtain the DOA and polarization estimates. In addition, to facilitate the above CNJD based framework, we proposed two Jacobi-type CNJD algorithms based on LU and LQ decompositions, respectively, and these two proposed CNJD algorithms are hence named LUCJD and LQCJD accordingly. With theoretical analysis, simulation verifications and comparisons, we give the following conclusions concerning the properties of the proposed strategy:

- The proposed CNJD based strategy does not require prior knowledge on the array’s spatial configuration. It could provide DOA and polarization estimates for at most six signals with distinct DOA’s, with an array of at least two EMVS’s. In addition, the CNJD problem involved in this strategy is neither Hermitian nor positive definite, and thus the many CNJD algorithms requiring Hermitianity and positive definiteness could not be used in our proposed scheme;
- Compared with parallel factor analysis (PARAFAC) based algorithms, the proposed LUCJD and LQCJD could provide improved accuracy of joint DOA and polarization estimation in colored noise, and low SNR levels. Contrarily, with almost white noise present and high SNR, the PARAFAC based methods are of more merits;
- Among all the compared CNJD algorithms (LUCJD, LQCJD, JTJD, FAJD, UWEDGE). The proposed LUCJD and LQCJD provide the best performance with regards to the accuracy of DOA and polarization estimation in all the simulated settings (with signal-to-noise ratio varying and with the noise covariance levels varying);
- Concerning the converging properties of the compared CNJD algorithms. We note that LQCJD is of very promising converging pattern, in which the performance index drops dramatically in the first 2∼3 iterations and becomes smooth within 10 iterations, under the considered simulation setting. In particular, LQCJD only slightly falls behind JTJD, yet largely outperforms UWEDGE, LUCJD, and FAJD, with regards to the undergone iterations. The other proposed CNJD method named LUCJD, however, is less efficient than most of the competitors.

## Figures and Tables

**Table 1.** The two proposed CNJD algorithms.

• **Input**: A set of square matrices ***C***_1_, ***C***_2_, ⋯, ***C****_K_* ∈ C^*L*×*L*^, and a threshold
• **Output**: The estimated unmixing matrix ***B̃***
• **Implementation:**
***B̃*** ← ***I***, *γ_old_* ← 0, *ζ* ← *τ*+1.
**while***ζ* ≥ *τ***do**
The U-stage or Q-stage: ***V*** ← ***I***
**for all** 1 ≤ *i* < *j* ≤ *L***do**
- *For U-stage in LUCJD*: obtain the optimal elementary upper-triangular matrix ***T***_(*i*,*j*)_ with its (*i*, *j*)th element determined by Equation (13)
*For Q-stage in LQCJD*: obtain the optimal elementary unitary matrix ***T***_(*i*,*j*)_ via Cardoso’s algorithm [45]
- Update matrices: ***V***←***T***_(*i*,*j*)_***V***, $C_{k}\leftarrow T_{\left(i,j\right)}C_{k}T_{\left(i,j\right)}^{H}$, *k* = 1, …, *K*
**end for**
The L-stage: ***L*** ← ***I***
**for all** 1 ≤ *j* < *i* ≤ *L***do**
- obtain optimal elementary lower-triangular matrix ***T***′_(*i*,*j*)_ with its (*i*, *j*)th element determined by Equation (13)
- Update matrices: ***V***←***T′***_(*i*,*j*)_***L***, $C_{k}\leftarrow {T^{\prime }}_{\left(i,j\right)}C_{k}{T^{\prime }}_{\left(i,j\right)}^{H}\left(k=1,\cdots ,K\right)$
**end for**
***B̃*** ← ***LVB̃***, $\gamma_{\mathrm{new}}\leftarrow {\left\|\mathrm{LV}-I\right\|}_{F}^{2}$, *ζ* ← |*γ_new_* − *γ_old_*|, *γ_old_* ← *γ_new_*
**end while**

**Table 2.** The proposed joint DOA and polarization estimation strategy.

**Input**: The EMVS array signals *X*(*t*) ∈ C^6×*N*^, and the number of sources *M* **Output**: The DOA estimates ***v***_(_*_θ̃_m__*_,_ *_φ̃_m__*_)_, and the polarization estimates *γ̃_m_*, *η̃_m_*, *m* =1, 2, …, *M* **Implementation:** - Calculate a set of auto(cross)-covariance matrices {***C****_l_*_,_*_k_* | *l*,*k* = 1, …, *N*} via Equation (5), and perform the proposed LUCJD or LQCJD algorithms (summarized in Table 1) on these matrices to obtain the estimate of the unmixing matrix ***B̃***. - Stack {***C****_l_*_,_*_k_* | *l*,*k* = 1, …, *N*} into a big matrix ***C*** ∈ C^36×*N*^2^^ by Equation (14), perform singular value decomposition (SVD) to ***C*** ∈ C^36×*N*^2^^, and obtain a noise subspace projector ***Π*** ∈ C^*N*^2^×*N*^2^^ by truncating the right SVD vectors according to the discussion below Equation (14). - Calculate ***μ****_l,k_* = diag(***B̃C****_l,k_****B̃****^H^*), and constructe a matrix ***Γ*** ∈ C^*N*^2^×6^ by Equation (15). Perform noise subspace projection, select the indices of ***Γ***’ columns that are associated with the smallest *M* noise subspace projection values into an index vector *λ* ∈ R*^M^* via Equation (16), and then use it to obtain the estimates for the angular-polarization steering vectors via ***p̃****_m_* = ***Ã***(;,*λ_m_*), where ***Ã*** = ***B̃***^−1^, and *m* =1, 2, …, *M*. - Estimate the source DOA’s and polarizations with {***p̃****_m_* | *m* =1,2,...,*M*} according to Equation (17)
